# Current Status of AMOEBA–IL: A Multipolar/Polarizable Force Field for Ionic Liquids

**DOI:** 10.3390/ijms21030697

**Published:** 2020-01-21

**Authors:** Erik Antonio Vázquez-Montelongo, José Enrique Vázquez-Cervantes, G. Andrés Cisneros

**Affiliations:** 1 Department of Chemistry, University of North Texas, Denton, TX 76201, USA; erik.vazquezmontelongo@unt.edu (E.A.V.-M.); enriquevazquezcervantes@my.unt.edu (J.E.V.-C.); 2 Center for Advanced Scientific Computing and Modeling (CASCaM), University of North Texas, Denton, TX 76201, USA

**Keywords:** ionic liquids, multipolar/polarizable force field, QM/MM, molecular dynamics, computational property prediction

## Abstract

Computational simulations of ionic liquid solutions have become a useful tool to investigate various physical, chemical and catalytic properties of systems involving these solvents. Classical molecular dynamics and hybrid quantum mechanical/molecular mechanical (QM/MM) calculations of IL systems have provided significant insights at the atomic level. Here, we present a review of the development and application of the multipolar and polarizable force field AMOEBA for ionic liquid systems, termed AMOEBA–IL. The parametrization approach for AMOEBA–IL relies on the reproduction of total quantum mechanical (QM) intermolecular interaction energies and QM energy decomposition analysis. This approach has been used to develop parameters for imidazolium– and pyrrolidinium–based ILs coupled with various inorganic anions. AMOEBA–IL has been used to investigate and predict the properties of a variety of systems including neat ILs and IL mixtures, water exchange reactions on lanthanide ions in IL mixtures, IL–based liquid–liquid extraction, and effects of ILs on an aniline protection reaction.

## 1. Introduction

The study of ionic liquid (IL) solutions by means of molecular dynamics (MD) or Monte Carlo (MC) computational simulations have become a useful tool to study these systems. These approaches can provide significant insights on structural, thermodynamic and transport properties. Many of these approaches employ classical force fields (FFs), which employ bonded (bond length, angle, etc.) and non–bonded (Coulomb, Van der Waals) terms to approximate the energy of these systems [1].

Several groups have developed pairwise additive force fields (FFs) for the simulation of ILs [2,3,4,5,6,7,8,9,10,11,12,13,14,15,16,17,18,19]. A large number of studies have been devoted to the modeling of ILs by MD based on these FFs [10,20,21,22,23,24,25,26,27,28,29,30,31,32,33,34,35,36,37,38,39,40,41,42,43,44,45,46,47,48,49,50,51,52,53,54,55,56,57,58,59,60,61,62,63,64,65,66]. In general, trends for thermodynamic, structural and transport properties are reproduced. However, the limited accuracy of current FFs results in over– or under–estimation of some of these properties. Case in point, errors in calculated enthalpies of vaporization compared to experiment may be as high as 50% [11,37]. One more issue that needs to be taken into account is the high viscosity of these liquids that results in the need for long simulation times to obtain converged data for transport properties.

Most FFs for ILs approximate the intermolecular electrostatic interaction by using a collection of fixed (generally atom–centered) point charges. This approximation results in the neglect of important effects including ***charge density anisotropy***, ***charge density overlap***, ***induction*** and other ***many body effects***. Some of these effects are explicitly or implicitly taken into account in advanced FFs such as AMOEBA.

The accurate reproduction of charge density anisotropy and electronic polarization is particularly important for highly charged systems such as ILs. Several methods have been developed to model polarization effects including the Drude oscillator [67,68], fluctuating charge [69,70] and induced dipole model [71,72,73,74]. However, care must be taken since FFs that use polarization fitted only on isolated molecules may not reproduce condensed–phase properties [75]. First principles calculations to determine partial charges for ILs have suggested that polarization and charge transfer effects play an important role in ILs [76,77]. One approach to implicitly introduce these effects involves the use of ab initio Born–Oppenheimer MD to calculate average partial charges for a variety of ILs, resulting in non–integer values on each ion. This approach has shown significant charge transfer occurs and calculated properties are in better agreement than integer atomic partial charges [60,78].

Long–range polarization effects have been show to be non–negligible for redox processes in ILs [79]. MD simulations by Yan et al. comparing a non–polarizable FF with a polarizable FF demonstrated that electronic polarizability is significant in IL systems [80]. Additionally, the inclusion of polarization effects in IL simulations yields more accurate property calculations when compared to experiment [81,82,83,84,85,86,87,88,89].

In particular, thermodynamic and structural properties are seen to be more accurate than for non–polarizable FFs as reported by Bedrov et al. [90]. For example, errors for the enthalpies of vaporization are observed to be substantially reduced, in some cases by 20–30% with the inclusion of polarization [86,89,90,91]. In addition, the inclusion of polarization makes the dynamics of ILs faster [86]. Although some transport properties may still pose a challenge, for example, errors for conductivity may be over 100% in some cases, which in some cases may also be due to the breakdown of the Nernst–Einstein equation [86]. A review on polarizable potentials for ILs has recently been published, which provides an overview of these effects/potentials, including a mention of some results from AMOEBA–IL [89].

In this review, we concentrate on the development and application of the multipolar/polarizable AMOEBA potential for ionic liquids, AMOEBA–IL. AMOEBA is implemented in several modeling packages including Tinker [92], Tinker–HP [93], Tinker/OpenMM [94], OpenMM [95], and AMBER (pmemd.gem) [96,97]. The next section, Section 2, describes the theoretical underpinning and computational approach for the parametrization of AMOEBA–IL. Section 3 and Section 4 present the application of AMOEBA–IL to investigate water exchange reactions on lanthanide cations and liquid–liquide benzene extraction in imidazolium–based IL mixtures. This is followed by the discussion of the appcliation of AMOEBA–IL to study a novel IL containing spyrocyclic pyrrolidinium in Section 5. Subsequently, the use of AMOEBA–IL to simulate the MM environment in the QM/MM investigation of an aniline protection reaction is presented in Section 6, followed by concluding remarks.

## 2. AMOEBA–IL Parametrization

AMOEBA is an advanced potential that aims to provide an accurate description of the energy of the target system. This is achieved by a series of bonded contributions including cross terms, as well as improved description of non–bonded interactions by means of atom–centered multipoles (up to quadrupole), explicit polarization, and the use of the buffered Halgren potential for Van der Waals interactions [72,98,99,100]. The polarization energy is calculated by means of induced atomic dipoles. Here, the induced dipoles are obtained by $\mu_{i;\alpha }^{ind}=\mu_{i}E_{i}$; where α is the atomic polarizability and $E_{i;\alpha }$ is the external electric field (generated by permanent multipoles and induced dipoles). The Tholé damping function is employed to avoid the so–called polarization catastrophe at short range [101].

(1) $$\begin{array}{lll} V_{\mathrm{AMOEBA}}\left(r^{N}\right) & = & \sum_{bonds}k_{i}^{bond}{\left(l_{i}-l_{i,0}\right)}^{2}\left[1+2.55\left(l_{i}-l_{i,0}\right)+3.793125{\left(l_{i}-l_{i,0}\right)}^{2}\right]\\ & + & \sum_{angles}k_{i}^{\theta }{\left(\theta_{i}-\theta_{i,0}\right)}^{2}[1+0.014\left(\theta_{i}-\theta_{i,0}\right)+5.6\times 10^{-5}{\left(\theta_{i}-\theta_{i,0}\right)}^{2}\\ & + & 7.0\times 10^{-7}{\left(\theta_{i}-\theta_{i,0}\right)}^{3}+2.2\times 10^{-8}{\left(\theta_{i}-\theta_{i,0}\right)}^{4}]\\ & + & \sum_{torsions}\frac{v_{n}}{2}\left(1+cos\left(n\omega -\gamma \right)\right)+\sum_{oop}0.02191414k_{\chi }\chi^{2}\\ & + & \sum_{str-bend}k_{sb}\left(b_{i}-b_{i,0}\right)\left(\theta_{i}-\theta_{i,0}\right)+\sum_{PI-tor}V_{PI-tor,i}+\sum_{tor-tor}V_{tor-tor,i}\\ & + & \frac{1}{2}\left[\sum_{mtp}V_{mtp,i}+\sum_{VdW}\epsilon_{ij}{\left(\frac{1+\delta }{\rho_{ij}+\delta }\right)}^{n-m}\left(\frac{1+\gamma }{\rho_{ij}^{m}+\gamma }-2\right)\right]+\sum_{pol}V_{pol,i} \end{array}$$

where the first five lines on the left hand side comprise the bonded terms, and the last line involves the non–bonded terms. The terms and parameters have been described in detail in References [72,98,99,100].

The parametrization philosophy of AMOEBA–IL is based on the accurate reproduction of quantum mechanical (QM) data for dimer and oligomer systems, as well as the reproduction of bulk properties. In all cases, the bonded terms have been taken from the original AMOEBA parameters where available. For the bonded AMOEBA parameters that have not been previously reported, torsional scans have been obtained at the MP2/6–311G(d,p) level [102].

All parameters for the non–bonded terms for AMOEBA–IL are fitted using counterpoise corrected QM inter–molecular interaction energies and QM energy decomposition analysis (EDA) for representative dimers [102,103,104,105,106]. The QM EDA data is employed to fit and compare the individual Coulomb, polarization and Van der Waals AMOEBA terms to reduce the error of each term. The original parametrization for the dimethyl imidazolium–based systems employed the restricted variational space (RVS) approach for the QM EDA reference using dimers in a single orientation at different distances [102,103].

**Figure 1:**
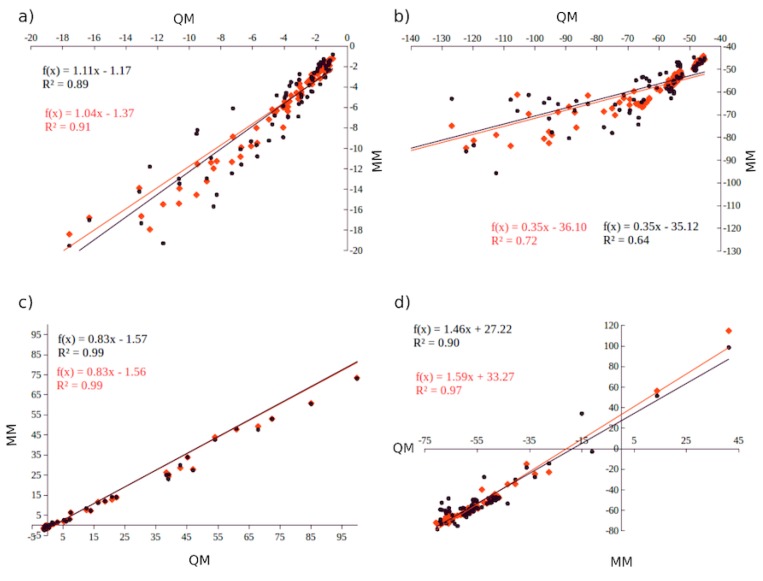
(**a**) polarization, (**b**) Coulomb, (**c**) Van der Waals and (**d**) Total inter–molecular interacion energies for 77 randomly oriented [sPyr${}^{+}$][BF${}_{4}$] dimers computed with AMOEBA–IL (MM) compared with QM EDA (SAPT) with (black), or without (red) inter–molecular polarization. Reproduced from Torabifard, H.; Reed, L.; Berry, M.T.; Hein, J.E.; Menke, E.; Cisneros, G.A. Computational and Experimental Characterization of a Pyrrolidinium-Based Ionic Liquid for Electrolyte Applications. *J. Chem. Phys.* **2017**, *147*, 161731. [105].

For the most recent parametrizations, the symmetry adapted perturbation theory (SAPT) method has been employed to obtain the reference components, coupled with randomly oriented molecules at different distances (see Figure 1) [105,106]. This newer approach takes advantage of an efficient implementation of SAPT in Psi4 [107], which enables the use of higher accuracy QM reference, as well as better representation of the dimer surface using randomly oriented dimers.

**Figure 2:**
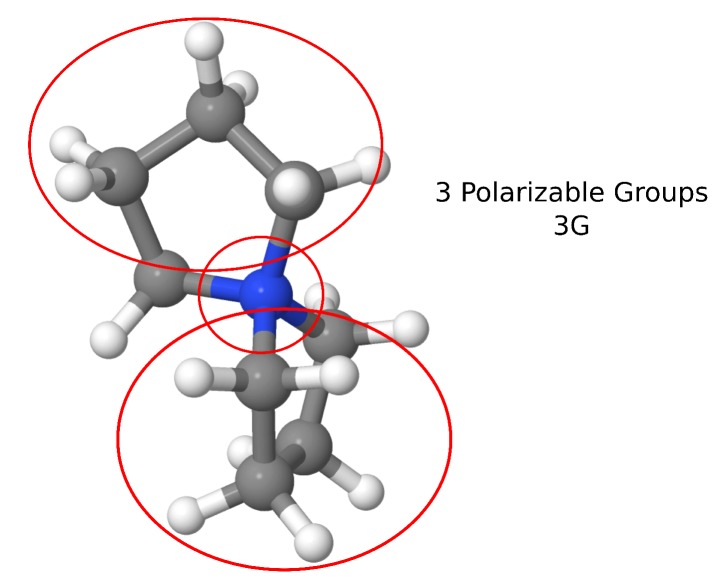
Spyrocyclic pyrrolidinium [SPyr] molecular structure, red circles roughly denote the intra–molecular polarization groups for the 3G set, from Torabifard, H.; Reed, L.; Berry, M.T.; Hein, J.E.; Menke, E.; Cisneros, G.A. Computational and Experimental Characterization of a Pyrrolidinium-Based Ionic Liquid for Electrolyte Applications. *J. Chem. Phys.* **2017**, *147*, 161731. [105].

As a specific example, one of the cations included in AMOEBA–IL is spyrocyclic pyrrolidinum [SPyr] (Figure 2). This cation comprises two five–membered rings fused by the quaternary ammonium atom. The two cycles are compsed of sp${}^{3}$ C atoms, which allow bending of the two rings with respect to the central N. The parametrization of the multipoles and VdW for this cation was performed using a set of thirty randomly–oriented [SPyr]–water dimers and validated against seventy [SPyr][BF${}_{4}$] randomly oriented dimers (Figure 1). Given the [SPyr] internal felxibility, two sets of parameters were developed comprising a set with no internal polarizable group (1G) and a set with three polarizable groups (3G) (Figure 2) to investigate the role of intra–molecular polarization (see below). The comparison of each non–bonded term with respect to QM EDA data provides a way to determine how each individual contribution is reproduced by AMOEBA–IL. This comparison allows a better understanding of the performance of the individual components of the force field.

Distributed atomic multipoles for AMOEBA can be obtained using the Gaussian distributed multipole analysis (GDMA) approach from Stone [108]. Alternatively, we have shown that strictly convergent distributed multipoles can be obtained from Hermite Gaussian functions using the Gaussian electrostatic model (GEM) fitting approach [109,110,111]. The Coulomb and polarization interactions are compared against the corresponding EDA counterparts. Additionally, it is possible to approximate intra–molecular polarization via polarizable groups as introduced by Ren and Ponder and employed for AMOEBA–IL (see Figure 1) [98,105].

The Van der Waals term is fitted by using the energies obtained by subtracting the AMOEBA calculated Coulomb and polarization from the total counterpoise–corrected QM energy [102,103,104,105]. Thus, the Van der Waals term effectively includes not only exchange and dispersion interactions, but also charge transfer effects and other errors in the non–bonded terms such as Coulomb penetration. In some cases it is advantageous to employ bulk properties to further refine the Van der Waals parameters [102,105,112].

This approach has resulted in parameters that provide accurate description of neat ILs and IL solutions (vide infra). For example, for [EMIM][EtSO${}_{4}$] the AMOEBA–IL calculated density and heat of vaporization at 298 K results in errors less than 1% [103], compared with errors ≈1.5% and >5% for density and ΔH${}_{vap}$ respectively with conventional point charges [113]. Similarly, for [EMIM][OTf], AMOEBA–IL results for these properties also show errors below 1% [104] compared with ≈1.5% for density and >1% compared with >7% for the heat of vaporization at 298 K [113,114]

## 3. Water Exchange Dynamics on Lanthanide Cations

Lanthanide ions (Ln-ions) are employed as contrast agents for biomedical imaging because of their luminescent and magnetic properties. One feature that can affect the contrast agent efficiency is the rate of ligand exchange on the complex, in particular that of water, [115,116], which can be modulated by altering the coordination environment. Thus, understanding the water exchange mechanism in Ln–ions can provide important insight for contrast agent development.

Some of us performed MD simulations employing AMOEBA to study the structure and dynamics on these solvent-exchange processes. The AMOEBA parameters for Gd${}^{3+}$, Dy${}^{3+}$, and Ho${}^{3+}$ ions were obtained by comparing energies calculated with the respective AMOEBA parameters with the interaction energies of Ln–water dimers obtained at the MP2/SDD/6-311G(d,p) level of theory (Stuttgart’s small core quasi-relativistic effective core potential (SDD) for Gd${}^{3+}$, Dy${}^{3+}$, and Ho${}^{3+}$ ions and 6-311G(d,p) basis sets for H and O atoms) and previously reported QM EDA data for different Ln–water dimers [117]. Polarizabilities of Lanthanide trivalent cations have been previously reported by Marjolin et al. [117,118,119].

**Figure 3:**
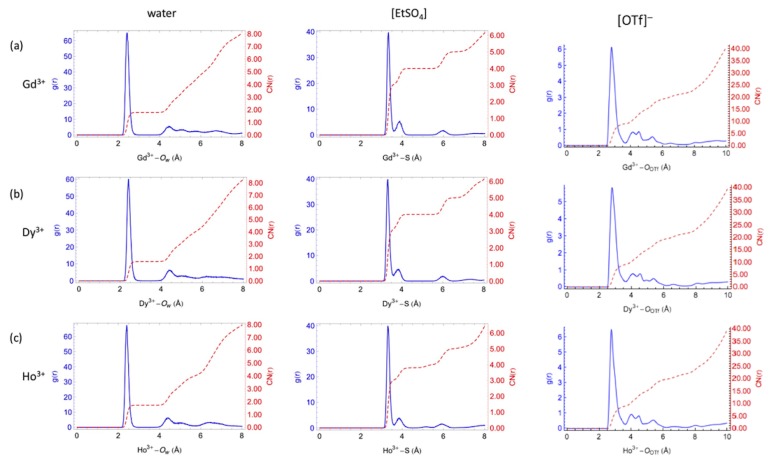
Radial distribution functions and integration curves of water (**left**) [EtSO${}_{4}$] (**center**) and [OTf] (**right**) around the lanthanide cations. Reproduced from Tu, Y.-J.; Allen, M.J.; Cisneros, G.A. Simulations of Water Exchange Dynamics on Lanthanide Ions in 1-Ethyl-3-Methylimidazolium Ethyl Sulfate ([EMIm][EtSO${}_{4}$]) and Water. *Phys. Chem. Chem. Phys* **2016**, *18*, 30323–30333. With permission from the PCCP Owner Societies [103], and Tu, Y.-J.; Lin, Z.; Allen, M.J.; Cisneros, G.A. Molecular Dynamics Investigation of Solvent-Exchange Reactions on Lanthanide Ions in Water/1-Ethyl-3-Methylimidazolium Trifluoromethylsulfate ([EMIm][OTf]). *J. Chem. Phys.* **2018**, *148*, 024503. Copyright 2018 American Physical Society.

The parameters for the lanthanide cations were tested by determining the coordination numbers and radial distribution functions (*g(r)*) in water and in the mixtures with the two ILs, and comparing to experimental results (see Figure 3). Consistent with experimental data, the Ln–Ow distances are seen to decrease as the ionic radius decreases from Gd${}^{3+}$ to Ho${}^{3+}$. Additionally, the AMOEBA–based simulations predict between eight and nine water molecules in the first hydration shell due to water-exchange events between the first and the second shells in agreement with experimental results [103].

In the water/[EMIm][EtSO${}_{4}$] system, Ln-ions can be surrounded by water and [EtSO${}_{4}$]${}^{-}$ anions (Figure 3). The two peaks observed in Figure 3 for the first coordination shell suggest the Ln–ions are all coordinated by four [EtSO${}_{4}$]${}^{-}$ anions. Three anions coordinate the respective lanthanide cation in a bidentate fashion, and one more as a monodentate ligand. Additionally, the first coordination shell of the Ln-ions contains one or two water molecules due to water-exchange events, resulting in the two observed peaks.

In the water/[EMIm][OTf] system, for for Gd${}^{3+}$, Dy${}^{3+}$, and Ho${}^{3+}$ ions, the Ln–O${}_{OTf}$g(r) patterns are similar. Ln-ions are coordinated with nine O atoms from six [OTf]${}^{-}$ anions in the first shell. The maxima for water/[EMIm][OTf] peaks are centered at shorter distances compared to water/[EMIm][EtSO${}_{4}$]. This difference indicates that the binding strength between the Ln-ions and water in water/[EMIm][OTf] is stronger compared to only water or water/[EMIm][EtSO${}_{4}$], possibly resulting from less steric repulsion between water and [OTf]${}^{-}$ than water–[EtSO${}_{4}$]${}^{-}$ and water–water in the first coordination shell.

The diffusion coefficients (D) of water ($D_{H_{2}O}$) and each Ln-ion ($D_{Ln}$) in water were calculated using Einstein’s relation (Equation (2)) [120], employing the slopes of mean-square displacement (MSD) as a function of time and compared to the corresponding values in water/[EMIm][EtSO${}_{4}$] and water/[EMIm][OTf]. The self-diffusivities of water in water/[EMIm][EtSO${}_{4}$] and water/[EMIm][OTf] are smaller than those in water because water molecules form strong hydrogen bonds with the [EtSO${}_{4}$]${}^{-}$ or [OTf]${}^{-}$ anions, which restrain the motion of water and lead to slower dynamics. Moreover, water molecules diffuse more rapidly in water/[EMIm][OTf] than in water/[EMIm][EtSO${}_{4}$] (1.33 × 10${}^{-8}$ to 1.94 × 10${}^{-8}$ cm${}^{2}$/s). The calculated ($D_{H_{2}O}$) and $D_{Ln}$ in water/[EMIm][EtSO${}_{4}$] and water/[EMIm][OTf] are consistent with the ${}^{17}$O-NMR experimental data [121,122,123,124].

(2) $$\begin{array}{c} 6Dt=\lim_{t\to \infty }\mathrm{MSD}\left(t\right) \end{array}$$

Water-exchange rates were calculated using the survival function method [125] (for water/[EMIm][EtSO${}_{4}$] system) and the direct method [126] (for water/[EMIm][OTf] system); both methods need a time parameter ($t^{*}$) for defining a real exchange event. A water-exchange event relates the time difference between a water molecule coming/leaving from the first solvation shell. In water, water-exchange rates exhibit the trend of Gd${}^{3+}$ > Dy${}^{3+}$ > Ho${}^{3+}$, but slower in water/[EMIm][EtSO${}_{4}$] and water/[EMIm][OTf]. The calculated water-exchange rates show the same trends as the ${}^{17}$O-NMR experiments, albeit they are slightly faster (Table 1). Notably, the water exchange rate (both experimental and calculated) in neat water is observed to decrease as the Ln–ion atomic number increases. Conversely, in both water/IL mixtures this trend is reversed, that is, the water exchange rate decreases with increasing atomic number of the Ln–ion.

**Figure 4:**
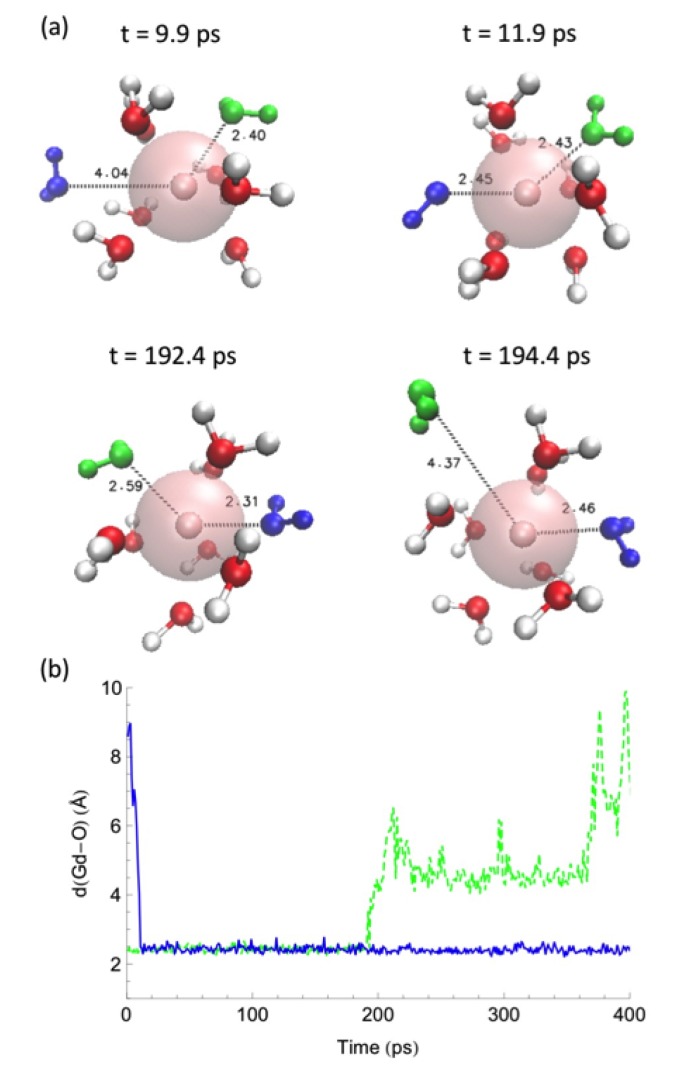
(**a**) Selected snapshots for a water exchange event on Gd${}^{3+}$ in water and (**b**) corresponding cation—water distance along the MD trajectory. A similar behavior is observed for Dy${}^{3+}$ and Ho${}^{3+}$ in water. Reproduced from Tu, Y.-J., Allen, M.J., Cisneros, G.A., (2016) “Simulations of Water Exchange Dynamics on Lanthanide Ions in 1-Ethyl-3-Methylimidazolium Ethyl Sulfate ([EMIm][EtSO${}_{4}$]) and Water”, *Phys. Chem. Chem. Phys.*, *18*, 30323–30333. With permission from the PCCP Owner Societies. [103].

**Figure 5:**
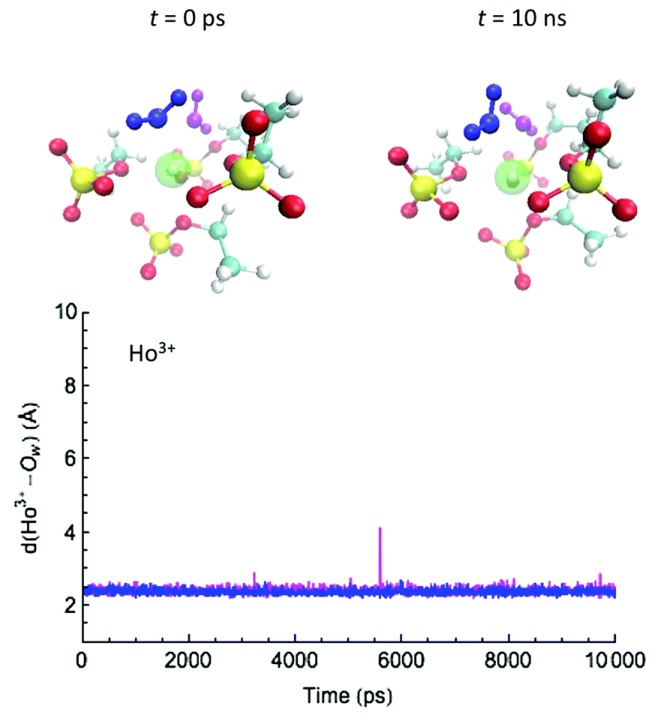
Selected snapshots for a water exchange event on Ho${}^{3+}$ in water/[EMIm][EtSO${}_{4}$] and corresponding cation–water distance along the MD trajectory. Similar behaviors were observed for Gd${}^{3+}$ and Dy${}^{3+}$. Reproduced from Tu, Y.-J., Allen, M.J., Cisneros, G.A., (2016) “Simulations of Water Exchange Dynamics on Lanthanide Ions in 1-Ethyl-3-Methylimidazolium Ethyl Sulfate ([EMIm][EtSO${}_{4}$]) and Water”, *Phys. Chem. Chem. Phys.*, *18*, 30323–30333. with permission from the PCCP Owner Societies [103].

**Figure 6:**
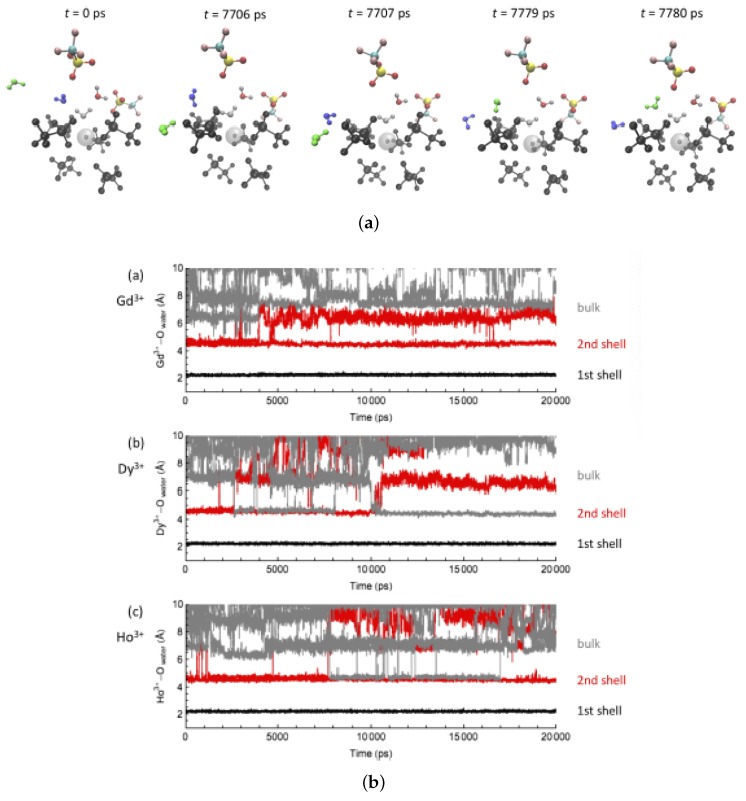
MD snapshots for a water-exchange event on Ho${}^{3+}$ during the simulation time, and the distance trajectories of the water O atoms with (**a**) Gd${}^{3+}$, (**b**) Dy${}^{3+}$, and (**c**) Ho${}^{3+}$ in water/[EMIm][OTf]. Reproduced from Tu, Y.-J.; Lin, Z.; Allen, M.J.; Cisneros, G.A. Molecular Dynamics Investigation of Solvent-Exchange Reactions on Lanthanide Ions in Water/1-Ethyl-3-Methylimidazolium Trifluoromethylsulfate ([EMIm][OTf]). *J. Chem. Phys.* **2018**, *148*, 024503. Copyright 2018 American Physical Society.

Water-exchange events between the first coordination shell and the bulk along the MD trajectories were analyzed by measuring the distance between the Ln–ion and the oxygen of the water molecules. (Figure 4, Figure 5 and Figure 6) in different solvent systems. In water, at the beginning of the simulation, the first hydration shell of Ln${}^{3+}$ has eight water molecules, forming a square antiprism (SAP) geometry. Along the simulation, one water molecule from the bulk joins the first hydration shell, and a nine-coordinate Ln-aquo complex is formed with a tricapped trigonal prism (TTP) geometry, followed by a water release to the outer hydration shells, and rearranging back to the SAP geometry. Based on their ionic radii (Gd${}^{3+}$ > Dy${}^{3+}$ > Ho${}^{3+}$), smaller Ln-ions form tighter aquo complexes with the neighboring water molecules, affecting the accessibility for the incoming water and thus impacting to the water-exchange rates. Therefore, our simulations suggest that the water exchange in neat water follows an associative mechanism based on the overlap of Ln-water distance trajectories for the incoming and outgoing water molecules in the first hydration shell.

For the water/[EMIm][EtSO${}_{4}$] system, initially the Ln${}^{3+}$ ion is coordinated with nine O atoms from four [EtSO${}_{4}$]${}^{-}$ and two water molecules. No exchanges of water or other ligands occurred in the first nanoseconds of MD simulations. Interestingly, [EtSO${}_{4}$]${}^{-}$ experienced rapid spin motions around the Ln-ion, resulting in the occasional increase of the Ln–water distance (dissociation of a water molecule), losing a water molecule and promoting a water-exchange process. The residence time trend of a water in the first shell is opposite to the one for water-exchange rates in water (Ho${}^{3+}$ < Dy${}^{3+}$ < Gd${}^{3+}$) and depends on the charge density of the Ln-ion. [EtSO${}_{4}$]${}^{-}$ anions bind strongly to smaller Ln-ions, increasing the steric effects in the first coordination shell, impeding the water/Ln${}^{3+}$ binding and resulting in a more facile release of water molecules from Ln${}^{3+}$. No water-exchange event was observed in all MD trajectories when two first shell water molecules were adjacent to each other at the beginning of the simulation. Based on these results, the water exchange mechanism for the water/[EMIm][EtSO${}_{4}$] mixture corresponds to a dissociative mechanism in contrast to the neat water mechanism.

For the water-exchange process in water/[EMIm][OTf] (Figure 6), at the beginning of the simulations, six [OTf]${}^{-}$ anions and one water molecule coordinated all Ln-ions in the first solvation shell. The [OTf]${}^{-}$ anions form a trigonal prism structure with a water molecule placed at one of the square faces, while the second solvation shell consists of two water molecules and two [OTf]${}^{-}$ anions, which undergo rapid rotational motions around the Ln-ion (similar to the first shell in the water/[EMIm][EtSO${}_{4}$] system). Occasionally, the second shell waters form hydrogen bonds with the O or F atoms of the first shell [OTf]${}^{-}$, ending in the dissociation of a water from the second shell to the bulk.

**Figure 7:**
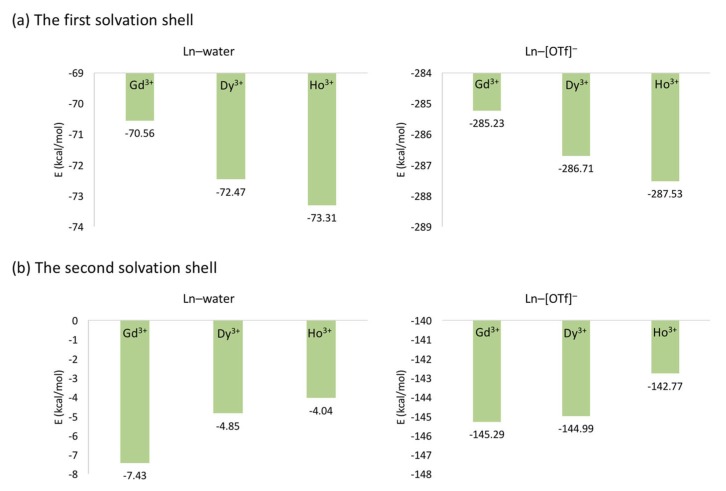
Average total interaction energies of lanthanide ions with a [OTf]${}^{-}$ anion and a first shell (**a**) and second shell (**b**) water. Reproduced from Tu, Y.-J.; Lin, Z.; Allen, M.J.; Cisneros, G.A. Molecular Dynamics Investigation of Solvent-Exchange Reactions on Lanthanide Ions in Water/1-Ethyl-3-Methylimidazolium Trifluoromethylsulfate ([EMIm][OTf]). *J. Chem. Phys.* **2018**, *148*, 024503. Copyright 2018 American Physical Society.

The average interaction energies of Ln-ion/[OTf]${}^{-}$ and Ln-ion/water (Figure 7) depend on the size of the Ln-ion. The large charge density of smaller Ln-ions in the first solvation shell result in a stronger binding for both [OTf]${}^{-}$ anion and water molecules. Conversely, this trend is opposite in the second solvation shell, possibly due to a larger screening effect.

Overall, the MD simulations are in agreement with experimental results with respect to water-exchange trends (In water, the rates decreases: Gd${}^{3+}$ > Dy${}^{3+}$ > Ho${}^{3+}$; in water/[EMIm][EtSO${}_{4}$] and water/[EMIm][OTf], the inverse trend is observed). MD-trajectory analysis indicates that in water, the water-exchange process is associative, due to stronger electrostatic interactions with the first shell water. Smaller ionic radius prevents the association of water with the first shell. On the other hand, in water/[EMIm][EtSO${}_{4}$] the process is dissociative. The dissociation of a water molecule from the first shell depends on the relative binding strength of the Ln-ion with [EtSO${}_{4}$]${}^{-}$ anions and water molecules. The smaller the Ln-ion, the stronger the anion binding, causing steric effects on the first shell water molecules. Albeit for water/[EMIm][OTf] the trend is similar to that in water/[EMIm][EtSO${}_{4}$], the water exchange process is different. The water-exchange event occurred between the second shell and bulk in water/[EMIm][OTf], and depends on the relative binding strength between the Ln-ion and the first shell [OTf]${}^{-}$ anions. The anion tightly binds to smaller Ln-ions, resulting in screening effect to the second shell water molecules, weakening the Ln-ion/water interactions, facilitating the water exchange process.

## 4. Liquid–liquid Extraction of Benzene from Dodecane–Benzene Mixture

Ionic liquids are attractive solvents for liquid-–liquid extraction due to their unique properties (low vapor pressure, reusability, thermal and chemical stability). Several researchers have obtained experimental evidence of the extraction of benzene (PhH) and other aromatic compounds from hydrocarbon mixtures like gasoline using ILs [127,128,129,130,131,132,133]. We have recently employed AMOEBA–IL to investigate the extraction of PhH from a gasoline–model using two imidazolium–based ILs [106].

**Figure 8:**
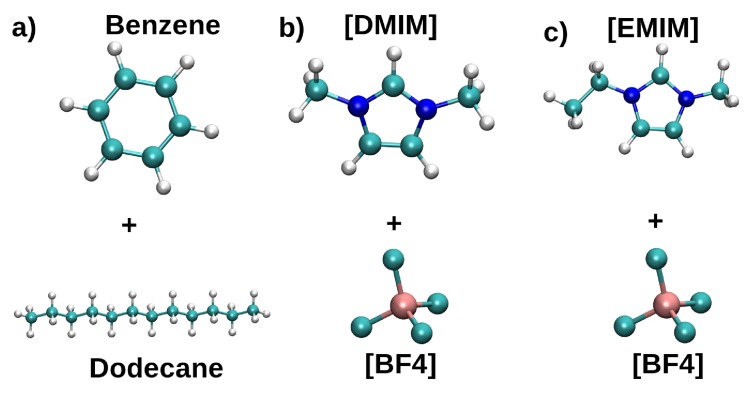
Schematic representation of molecules used for the simulation of benzene extraction from gasoline with ILs. (**a**) Benzene (PhH) and dodecane (NC12), (**b**) [DMIM][BF${}_{4}$], and (**c**) [EMIM][BF${}_{4}$] Reproduced from Vazquez-Montelongo, E.A.; Cisneros, G.A.; Flores–Ruiz, H.M. Multipolar/Polarizable Molecular Dynamics Simulations of Liquid-Liquid Extraction of Benzene from Hydrocarbons Using Ionic Liquids. *J. Mol. Liq.* **2019**, doi:10.1016/j.molliq.2019.111846, [106].

Two systems consisting of a gasoline–model (1:1 mixture of n-dodecane, NC12, and PhH) and 1,3-dimethylimidazolium tetrafluorobrorate, [DMIM][BF${}_{4}$], or ethyl-methylimidazolium tetrafluorobrorate, [EMIM][BF${}_{4}$] as extracting agents were considered (Figure 8). The density profiles along the z direction were used to measure the PhH extracting capabilities, and the spatial distribution functions (SDF) were employed to gain further insights on the interactions within the studied mixtures.

**Figure 9:**
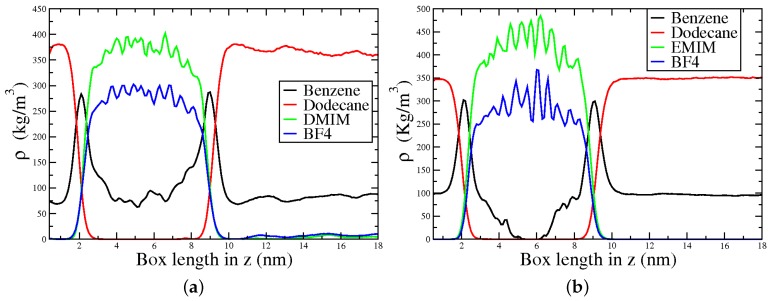
Density profile along the *z* direction for the (**a**) ternary mixture [DMIM][BF${}_{4}$]/benzene/dodecane and (**b**) ternary mixture [EMIM][BF${}_{4}$]/benzene/dodecane. Reproduced from Vazquez-Montelongo, E.A.; Cisneros, G.A.; Flores–Ruiz, H.M. Multipolar/ Polarizable Molecular Dynamics Simulations of Liquid-Liquid Extraction of Benzene from Hydrocarbons Using Ionic Liquids. *J. Mol. Liq.* **2019**, doi:10.1016/j.molliq.2019.111846, [106].

Figure 9a shows that a fraction of PhH goes to the IL rich region in the [DMIM][BF${}_{4}$] system. Conversely NC12 remains in the hydrocarbon rich one, suggesting a poor affinity of NC12 for [DMIM][BF${}_{4}$], consistent with experimental and computational results using other ILs. On the other hand, for [EMIM][BF${}_{4}$] (Figure 9b), a smaller amount of PhH is extracted, resulting in a region between 5–6 nm with no PhH. Additionally, NC12 does not have affinity with [EMIM][BF${}_{4}$].

**Figure 10:**
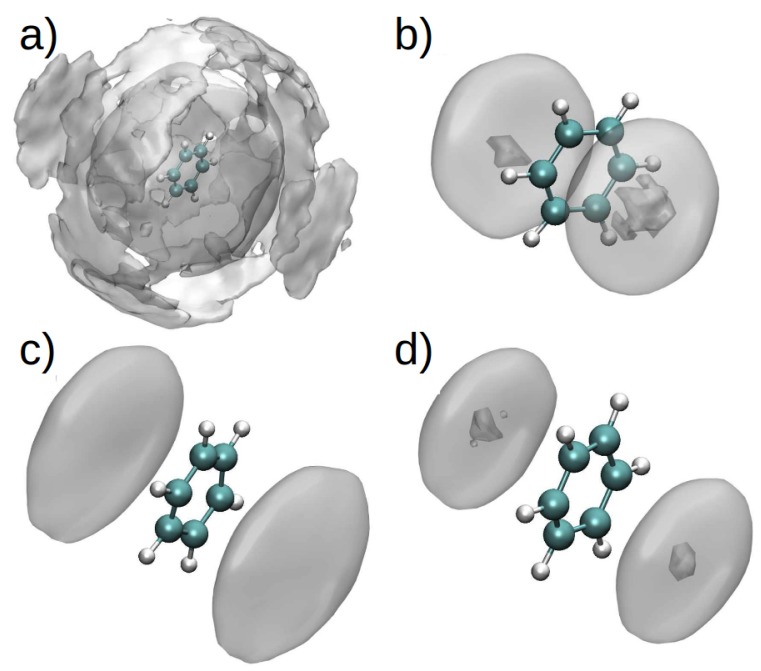
(**a**) SDF of PhH and one nitrogen atom in the ring of [DMIM] in a binary mixture (PhH-[DMIM][BF4]), (**b**) difference between the SDF of PhH-[DMIM] in the binary mixture and the SDF of PhH and one nitrogen atom in the ring of [DMIM] in the ternary mixture (PhH-NC12-[DMIM][BF4]). (**c**) SDF of PhH and one nitrogen atom in the ring of [EMIM] in a binary mixture (PhH-[EMIM][BF4]), (**d**) difference between the SDF of PhH-[EMIM] in the binary mixture and the SDF of PhH and one nitrogen atom in the ring of [EMIM] in the ternary mixture (PhH-NC12-[EMIM][BF4]). Reproduced from. Reproduced from Vazquez-Montelongo, E.A.; Cisneros, G.A.; Flores–Ruiz, H.M. Multipolar/Polarizable Molecular Dynamics Simulations of Liquid-Liquid Extraction of Benzene from Hydrocarbons Using Ionic Liquids. *J. Mol. Liq.* **2019**, doi:10.1016/j.molliq.2019.111846, [106].

The spatial distribution function (SDF) for PhH/[DMIM]${}^{+}$ and PhH/[EMIM]${}^{+}$ interactions in binary systems (Figure 10a,c), show that at short range, there are stacking interactions between aromatic groups, and these interactions are similar in binary and ternary systems (Figure 10b,d). On the other hand, at long range, there are more PhH/[DMIM]${}^{+}$ interactions compared with PhH/[EMIM]${}^{+}$. These differences arise from the imidazolium ion’s alkyl chain length.

**Figure 11:**
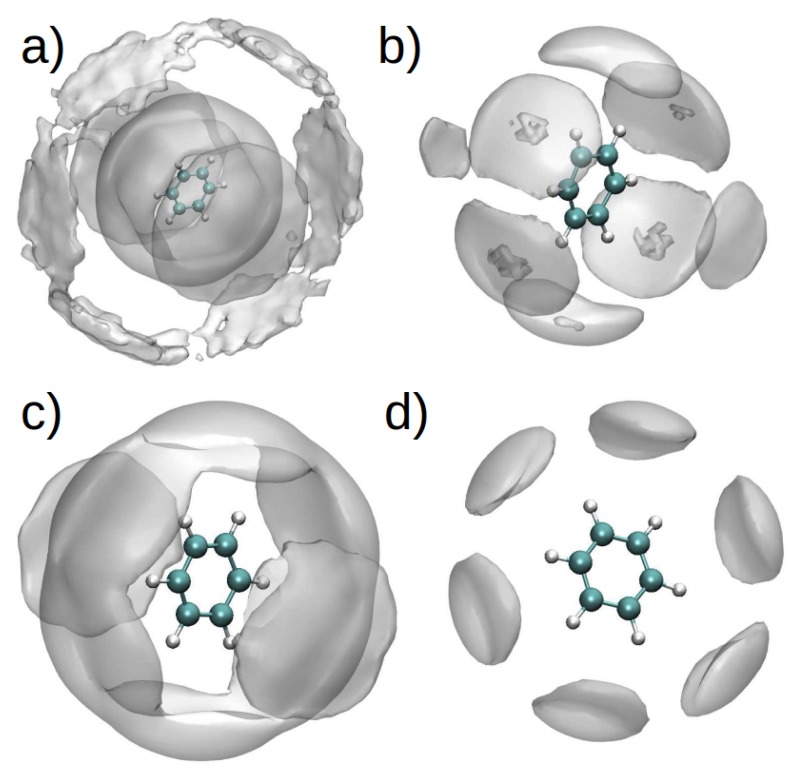
(**a**) SDF of PhH and the boron atom in [BF4], in a binary mixture (PhH-[DMIM][BF4]), (**b**) difference between the SDF of PhH and [BF4] in the binary mixture and the SDF of PhH and boron atom in [BF4] in the ternary mixture (PhH-NC12-[DMIM][BF4]). (**c**) SDF of PhH and the boron atom in [BF4], in a binary mixture (PhH-[EMIM][BF4]), (**d**) difference between the SDF of PhH and [BF4] in the binary mixture, and the SDF of PhH and boron atom in [BF4] in the ternary system (PhH-NC12-[EMIM][BF4]). Reproduced from Vazquez-Montelongo, E.A.; Cisneros, G.A.; Flores–Ruiz, H.M. Multipolar/Polarizable Molecular Dynamics Simulations of Liquid-Liquid Extraction of Benzene from Hydrocarbons Using Ionic Liquids. *J. Mol. Liq.* **2019**, doi:10.1016/j.molliq.2019.111846, [106].

By contrast, the SDF for the PhH/[BF${}_{4}$] interactions in both binary systems (Figure 11a,c) did not exhibit significant differences, indicating affinity between the anion and PhH. Additionally, [BF${}_{4}$]${}^{-}$ is observed to interact with PhH in the [EMIM][BF${}_{4}$] ternary system only along the plane of the PhH molecule, whereas [BF${}_{4}$]${}^{-}$ interacts with PhH in the [DMIM][BF${}_{4}$] ternary system both along the edge and plane of the ring (Figure 11b,d).

These results suggest that the interactions of both anion and cation increase the selectivity for PhH by [DMIM][BF${}_{4}$] as extracting agent, compared with [EMIM][BF${}_{4}$]. One possible explanation of this behaviour is due to the symmetry of the [DMIM]${}^{+}$, which may allow a closer arrangement for both cation and anions in the IL–rich phase, and thus more favorable arrangement of the anions around the PhH in the IL–rich phase.

## 5. Computational/Experimental Characterization of Spyrocyclic Pyrrolidinium/Tetrafluoroborate [Spyr][BF${}_{4}^{-}$]

ILs have been studied as possible electrolytes of lithium-ion batteries in order to avoid several safety concerns present when organic electrolytes are used. Unfortunately, most tested IL pairs have been proven to be poor electrolytes in batteries [134,135]. One possible approach to improve specific IL ion transfer performance for the design of electrolyte-electrode couples in batteries includes a deeper understanding of thermodynamic and transport properties at the atomic-level. For that reason, computational simulations may be used not only to study these systems, but also to predict their properties, to narrow the wide variety of possible cation-anion combinations.

Some of us employed AMOEBA–IL to investigate the properties of an IL pair as a possible electrolyte candidate involving spyrocyclic pyrrolydinium ([SPyr]) combined with [BF${}_{4}^{-}$] both neat and in a mixture with 10% Li${}^{+}$. The computational studies were coupled with experimental synthesis and characterization of the neat [sPyr${}^{+}$][BF${}_{4}^{-}$]. To our knowledge, this was the first literature report of an example of a pyrrolidinium–based cation comprising a quaternary amonium group bearing two four-membered carbon cycles.

**Figure 12:**
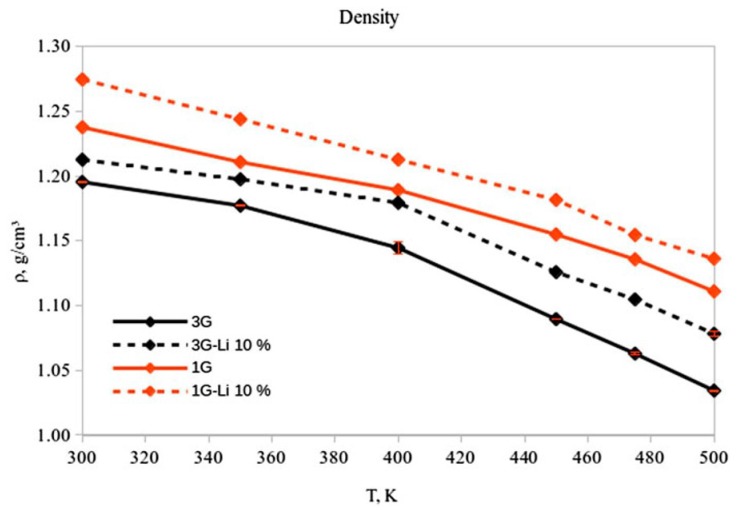
Calculated density at different temperatures for [sPyr${}^{+}$][BF${}_{4}^{-}$] using one and three polarizable groups for 300–500 K. Reproduced from Torabifard, H.; Reed, L.; Berry, M.T.; Hein, J.E.; Menke, E.; Cisneros, G.A. Computational and Experimental Characterization of a Pyrrolidinium-Based Ionic Liquid for Electrolyte Applications. *J. Chem. Phys.* **2017**, *147*, 161731 [105].

The density of pure [sPyr${}^{+}$][BF${}_{4}^{-}$] was calculated for a range of temperatures between 300 to 500 K using the set parameters for one (1G) and three (3G) polarizable groups (Figure 2), as explained in Section 2. A decrease between 4%–8% can be observed in densities calculated considering intra-molecular polarization in the force field (3G) (Figure 12). Interestingly, the comparison between densities using 1G and 3G parameter sets shows a decrease of 12.7% and 16.1%, respectively, in a non linear fashion from 300 to 500 K. The 1G parameter set shows a density of 1.19 g/cm${}^{3}$ and a volume of 297.45 Å${}^{3}$ at 400 K, and 1.15 g/cm${}^{3}$ (303.31 Å${}^{3}$) at 450 K. On the other hand, for the 3G set of parameters, the densities and volumes at 400 K and 450 K were 1.14 g/cm${}^{3}$ (324.70 Å${}^{3}$) and 1.09 g/cm${}^{3}$ (308.12 Å${}^{3}$), respectively.

The mixture of these IL with 10% Li was also simulated. In general, the calculated densities for the mixture with both set of parameters are approximately 2%–4% higher than the density in the neat IL at the same range of temperatures (Figure 12). These results are consistent with densities obtained experimentally for other neat and Li/IL mixtures [136,137,138,139]. Similar to the neat IL, a noticeable change in density for the 1G and 3G systems (5.4% and 3.1%, respectively) was observed in the mixture between 400 and 450 K. For the 1G set of parameters at 400 K, the density (volume) correspond to 1.21 g/cm${}^{3}$ (275.06 Å${}^{3}$), compared with 1.18 g/cm${}^{3}$ (282.31 Å${}^{3}$) at 450 K. The density(volume) for the 3G system at 400 K are 1.18 g/cm${}^{3}$ (282.84 Å${}^{3}$), whilst at 450 K the corresponding properties are 1.13 g/cm${}^{3}$ (296.31 Å${}^{3}$).

The self–diffusion coefficients were also calculated for the same range of temperatures. The self-diffusion coefficients for the 1G system exhibits an increase of 5.6% in $D_{\pm }$ from 300 K to 500 K, which is not considered a significant change compared with the $D_{\pm }$ = 65.1% observed with the inclusion of intra-molecular polarization (3G) from 300 K to 500 K. These results suggest that a more accurate description of many body interactions speeds up the diffusion of the ions in the system.

From 300–400 K, the 3G parameter set does not show a significant change in the self-diffusion coefficient (<1.5%), while an increment in $D_{\pm }$ of 15.3% from 400 K to 500 K is observed. Conversely, the diffusion coefficient changes when intra–molecular polarization is neglected (1G) are not significant (1.7%). These results are consistent with the expected self-diffusion coefficients for smaller cations, showing that the bigger size of the [sPyr${}^{+}$] cation results in slower diffusion.

On the other hand, for the 10% Li${}^{+}$ mixture, the ions diffuse faster using the 1G parameter set: for temperatures between 450 and 500 K there is an increment in the diffusion coefficient with respect the neat IL. The mixture with intra-molecular polarization (3G model), shows that self-diffusion coefficient decreased at the same range of temperatures compared to neat IL 3G model.

The observed differences in calculated results between the 1G and 3G parameter sets for both the neat and Li mixtures are due the shortcomings of the 1G model in describing the change in charge density distribution caused by the changes in the internal structure of SPyr, specifically the bending of the cycles. Conversely, the 3G set enables the response of the polarization due to changes in the cation structure, providing a better representation of the inter–molecular interactions in the system.

**Figure 13:**
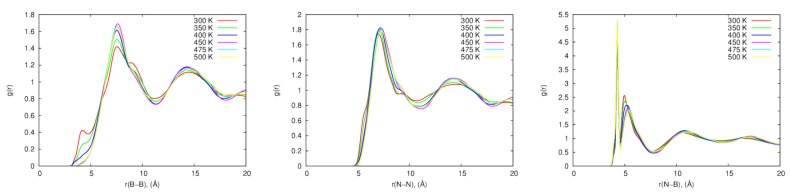
Radial distribution functions for [sPyr${}^{+}$][BF${{}_{4}}^{-}$] with three polarizable groups (3G) at different temperatures. Reproduced from Torabifard, H.; Reed, L.; Berry, M.T.; Hein, J.E.; Menke, E.; Cisneros, G.A. Computational and Experimental Characterization of a Pyrrolidinium-Based Ionic Liquid for Electrolyte Applications. *J. Chem. Phys*. **2017**, *147*, 161731, [105].

Radial Distribution Functions for anion-anion (B-B), anion-cation (B-N), and cation-cation (N-N) for both sets of parameters were calculated between 300 and 500 K as shown in Figure 13. The B-B RDF shows a small peak at 4 Å at 300, 350 and 400 K, which can be explained by the proximity of the [BF${}_{4}^{-}$] ions at those temperatures. Interestingly, this peak vanished at higher temperatures for the 3G model. The N-N RDF shows a plateau at 9.5 Å for the 300-400 K range, but it also vanished at higher temperatures. These differences support the hypothesis that the inter–ionic interactions are stronger with the inclusion of the intra-molecular polarization in this system. Additionally, the differences in RDFs at different temperatures, especially the loss of the peak for the B-B RDF at close distance, suggest a difference in the ordering of the ions at low temperatures compared with the high temperature systems.

**Figure 14:**
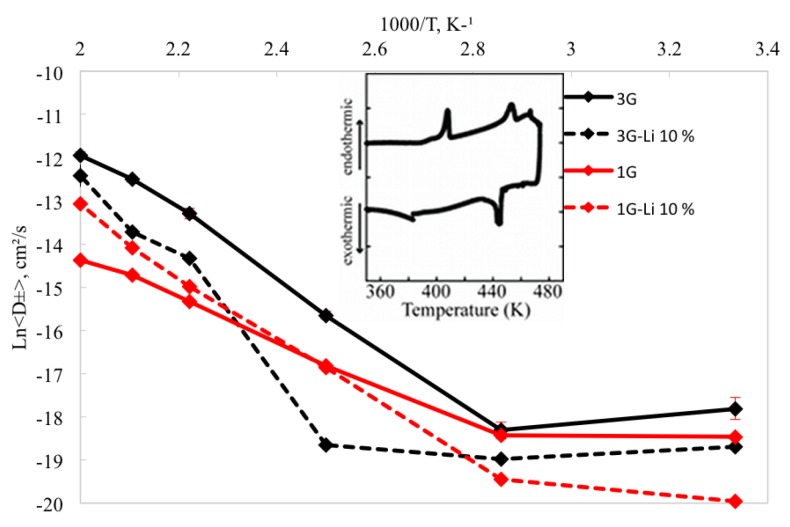
Calculated diffusion coefficients at different temperatures for [sPyr${}^{+}$][BF${}_{4}^{-}$] using one (1G) and three (3G) polarizable groups with and without 10% Li${}^{+}$. Reproduced from Torabifard, H.; Reed, L.; Berry, M.T.; Hein, J.E.; Menke, E.; Cisneros, G.A. Computational and Experimental Characterization of a Pyrrolidinium-Based Ionic Liquid for Electrolyte Applications. *J. Chem. Phys.* **2017**, *147*, 161731 [105].

The above results suggest a significant change in thermodynamic and transport properties at temperatures between 400 K to 450 K. It should be noted that the computational results were obtained prior to the experimental synthesis and characterization of the neat [sPyr${}^{+}$][BF${{}_{4}}^{-}$]. Subsequent to the observation in the significant changes in RDFs (and other properties) between 400 and 450 K, the neat IL pair was synthesized and differential scanning calorimetry (DSC) was performed. The inset in Figure 14 corresponds to the experimental DSC thermogram, which shows that this IL pair has a melting point of 448 K, enthalpy of fusion of 181 J/g, crystalization onset of 446 K and enthalpy of crystalization of 350 J/g. Thus, the calculated structural (and thermodynamic/transport) changes observed between 400 and 450 K are in very good agreement with the experimental results.

## 6. QM/MM Simulation of an Aniline Protection Reaction in H${}_{2}$O/[EMIm][BF${}_{4}$]

AMOEBA–IL has also been applied to study chemical reactions in IL mixtures. In particular, a sample organic reaction of the *N-tert*-butoxycarbonylation of aniline in an IL mixture by polarizable QM/MM was recently performed [140]. To our knowledge, this is the first example of a QM/MM simulation of a reaction in ILs using multipolar/polarizable methods for the classical environment. This is an example of an important reaction to control functional groups in the synthesis of drug molecules, also known as *N-t*-Boc reactions [141]. Several reactions mechanisms, considering the effects of ionic liquids (water/1-ethyl, 3-methyl imidazolium/tetrafluoroborate ([EMIm][BF${}_{4}$])/water mixture) as solvents via QM/MM simulations were investigated. The reaction mechanisms were characterized by performing minimum energy path (MEP) optimizations using two different chain–of–replica methods: the quadratic string method (QSM) and the nudged elastic band (NEB) [112,142].

**Figure 15:**
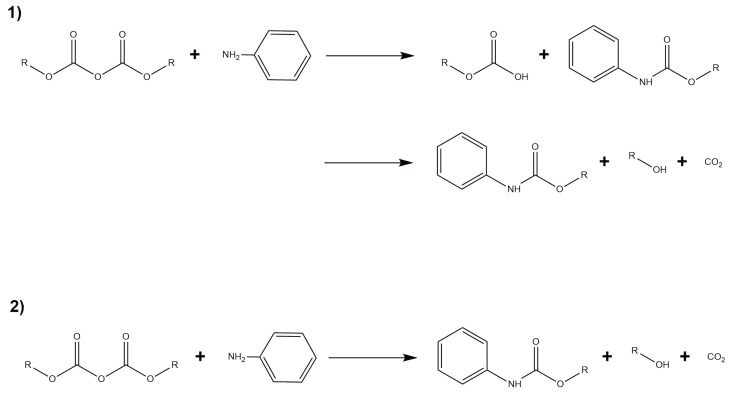
Reaction scheme for the *N-tert*-butoxycarbonylation of aniline. Panel (**1**) describes the step–wise mechanism and panel (**2**) describes the concerted mechanism mentioned above.

Depending on which group is attacked, the reaction may take place via two different mechanisms (see Figure 15): The first corresponds to a step–wise mechanism, where the nucleophilic attack does not happen concommitantly with the formation of CO${}_{2}$. The second mechanism proceeds via an concerted path where CO${}_{2}$ is formed at the same time as the nucleophilic attack occurs.

These two possible mechanisms were studied using two different configurations for the system: configuration 1 was obtained by restraining the distance of one IL ion pair to the solute (aniline and BoC) in the QM/MM optimization of snapshots taken from MD simulations. This configuration includes 149 atoms in the QM region.

**Figure 16:**
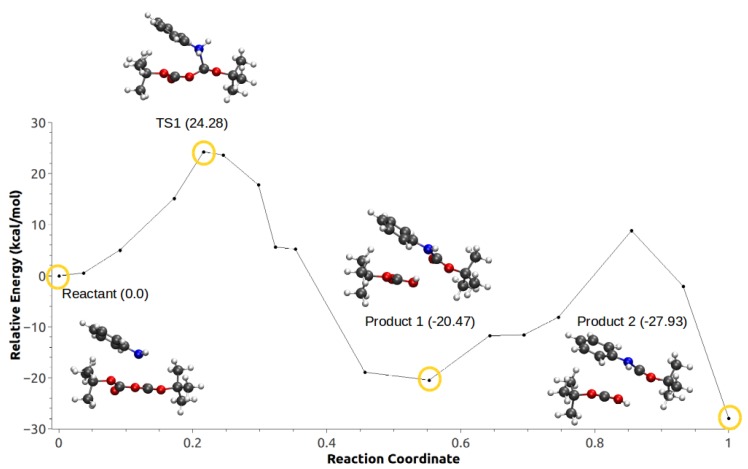
Minimum energy path for configuration 1, mechanism 1. Reproduced from Vazquez-Montelongo, E.A.; Vazquez-Cervantes, J.E.; Cisneros, G.A. Polarizable ab initio QM/MM Study of the Reaction Mechanism of N-tert-Butyloxycarbonylation of Aniline in [EMIm][BF${}_{4}$]. *Molecules* **2018**, *23*, 2830, doi:10.3390/molecules23112830 [140].

Configuration 2 was obtained based on QM/MM optimization of a series of snapshots from unrestrained MD simulations where the IL anions and cations are not close to the solvent. Configuration 2 includes 155 atoms in the QM subsystem. Both configurations were investigated in water/IL QM/MM systems. In the first mechanism, the nucleophilic attack in configuration 1 is followed by a proton transfer from the aniline to the Boc group, allowing the formation of *tert*-butyl carbamate and *tert*-butyl carboxylic acid as products (Figure 16).

**Figure 17:**
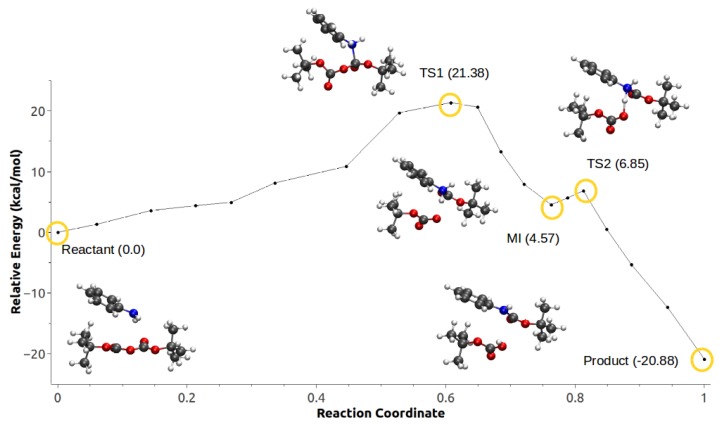
Minimum energy path for configuration 2, mechanism 1. Reproduced from Vazquez-Montelongo, E.A.; Vazquez-Cervantes, J.E.; Cisneros, G.A. Polarizable ab initio QM/MM Study of the Reaction Mechanism of N-tert-Butyloxycarbonylation of Aniline in [EMIm][BF${}_{4}$]. *Molecules* **2018**, *23*, 2830, doi:10.3390/molecules23112830.

For configuration 2, the CO${}_{2}$ is formed concurrently with the carbamate. The energy barrier in this case is roughly 3 kcal/mol lower compared with configuration 1. Fluctuations in anisotropic field due to the presence of IL pairs stabilize a metastable intermediate (MI) produced by the nucleophilic attack, coupled with a second low-barrier TS corresponding to the proton transfer followed by the formation of the products (Figure 17).

**Figure 18:**
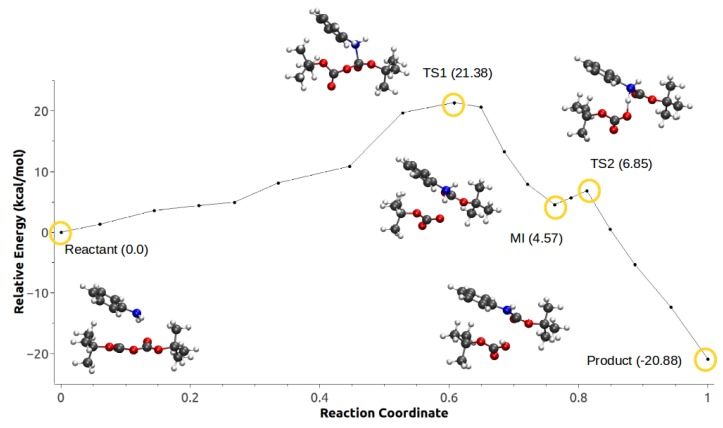
Minimum energy path for configuration 2, mechanism 2. Reproduced from Vazquez-Montelongo, E.A.; Vazquez-Cervantes, J.E.; Cisneros, G.A. Polarizable ab initio QM/MM Study of the Reaction Mechanism of N-tert-Butyloxycarbonylation of Aniline in [EMIm][BF${}_{4}$]. *Molecules* **2018**, *23*, 2830, doi:10.3390/molecules23112830 [140].

In the second mechanism, the minimum energy path for configuration 1 did not converge. On the other hand, configuration 2 shown in Figure 18 has a rate-limiting step barrier similar to the barriers of the configurations in mechanism 1, corresponding to the nucleophilic attack step. It also shows a (MI) previous to a second energy barrier that corresponds to the proton transfer of the aniline to the Boc groups that leads to the formation of *tert*-butanol, CO${}_{2}$ and *tert*-butyl carbamate.

**Figure 19:**
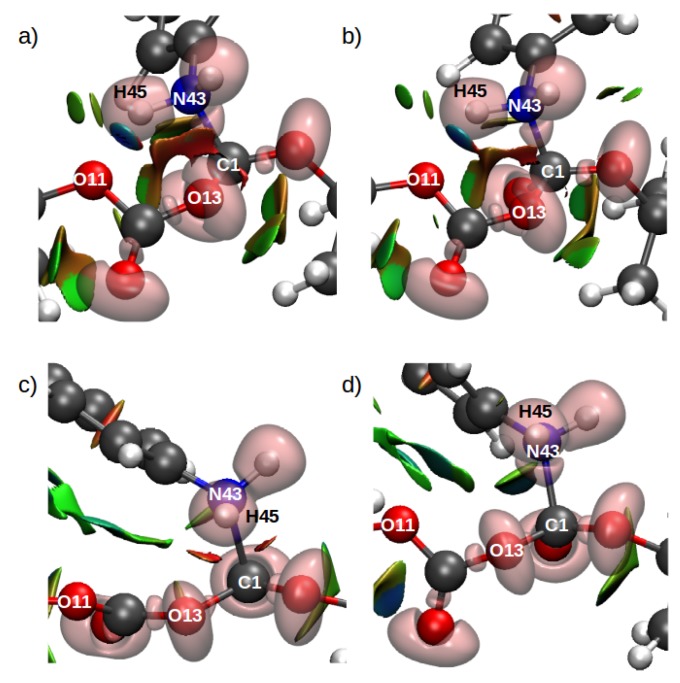
Combined ELF/NCI surfaces for the TS structures for the rate limiting step in Scheme c. (**a**) gas–phase, (**b**) di–chloromethane (implicit solvent), (**c**) configuration C1, (**d**) configuration C2. The isovalues for ELF is 0.83 and for NCI is 0.5 with a color scale of −0.05 au < sign(λ2)ρ < 0.05 au. Reproduced from Vazquez-Montelongo, E.A.; Vazquez-Cervantes, J.E.; Cisneros, G.A. Polarizable ab initio QM/MM Study of the Reaction Mechanism of N-tert-Butyloxycarbonylation of Aniline in [EMIm][BF${}_{4}$]. *Molecules* **2018**, *23*, 2830, doi:10.3390/molecules23112830 [140].

**Figure 20:**
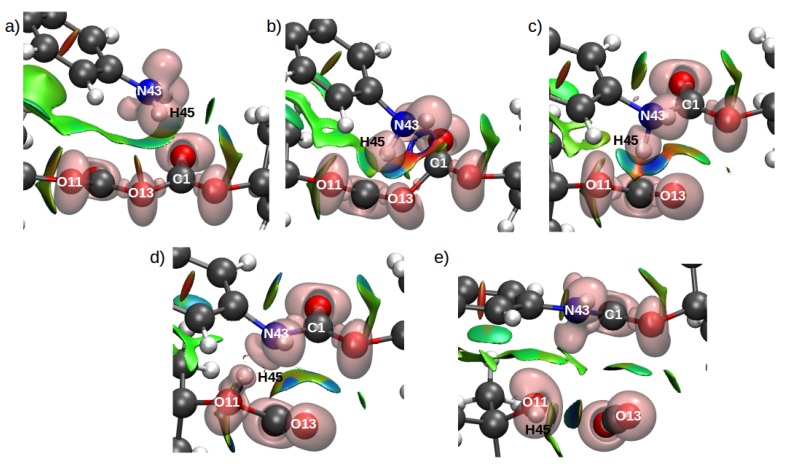
Combined ELF/NCI surfaces of the critical structures for configuration C2 for mechanism 2. Panels (**a**–**e**) show the reactants, TS1, MI, TS2 and products structures, respectively. The isovalues for ELF is 0.83 and for NCI is 0.5 with a color scale of −0.05 au < sign(λ2)ρ < 0.05 au. Reproduced from Vazquez-Montelongo, E.A.; Vazquez-Cervantes, J.E.; Cisneros, G.A. Polarizable ab initio QM/MM Study of the Reaction Mechanism of N-tert-Butyloxycarbonylation of Aniline in [EMIm][BF${}_{4}$]. *Molecules* **2018**, *23*, 2830, doi:10.3390/molecules23112830 [140].

Analysis of the electronic wavefunctions via combined electron localization function (ELF) and non-covalent interaction index (ELF/NCI) were performed to obtain more insights related to the evolution of the intra- and intermolecular interactions in the system. The ELF/NCI analyses for the TS structures for both configurations for the rate-limiting step in mechanism 1 (Figure 19) shows a disynaptic basin between the C1 and N43 which suggests the formation of a covalent bond between these two atoms in the TS. This means that in all cases, the TS structure corresponds to the mentioned nucleophilic attack and is part of a late TS. By contrast the TS structures for the second mechanism in both configurations did not show a disynaptic basin between N43 and C1. Nevertheless, a strong attractive interaction in the form of a ring can be observed in Figure 20b.

These results indicate a stabilization of an early TS provided by the solvent environment. Furthermore Figure 20c shows a bifurcated H-bond interaction between H45, O11 and O13 which explains the transfer of H49 to O11 in TS2. The NCI analyses did not show surfaces between H45 and N43 in TS2 which suggests a longer distance between the products.

**Figure 21:**
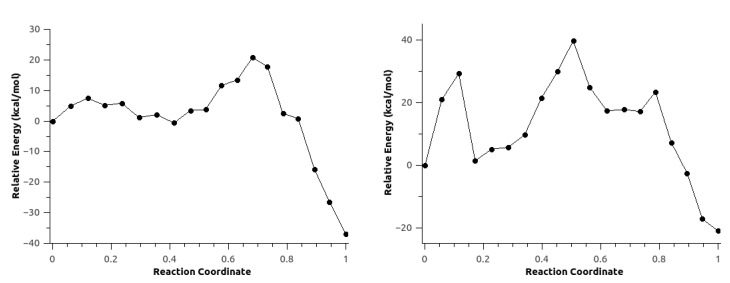
Minimum energy path for Scheme c (**right**) and d (**left**) for configuration C2 without the AMOEBA polarization term. Reproduced from Vazquez-Montelongo, E.A.; Vazquez-Cervantes, J.E.; Cisneros, G.A. Polarizable ab initio QM/MM Study of the Reaction Mechanism of N-tert-Butyloxycarbonylation of Aniline in [EMIm][BF${}_{4}$]. *Molecules* **2018**, *23*, 2830, doi:10.3390/molecules23112830 [140].

Finally, MEPs for both mechanisms using configuration 2 were re-optimized neglecting the polarization of the classical region to investigate the effect of the polarization of the environment on the reaction path. The first mechanism resulted in an energy profile similar to the one obtained with explicit polarization. However, this MEP did not show the second TS corresponding to the proton transfer. The second mechanism showed an energy profile with some irregularities suggesting a rearrangement of environmental solvent molecules The energy barrier for this mechanism was almost twice the value of the MEP shown in Figure 18. The similarities observed between the energy profiles with and without explicit polarization presented in Figure 21 can be explained because of the use of the optimized path with polarization as initial guess for the MEP optimization without polarization, considering also that the optimized path corresponded to the potential energy surface rather than free energy surface.

These results show that the *N-tert*-butoxycarbonylation of aniline can take place via either concerted or sequential reaction mechanism, depending on the chosen configuration for the ionic pair. Overall, the rate-limiting step for this reaction corresponds to the nucleophilic attack of the aniline. The products for each mechanism depend on which Boc group is attacked for the aniline. ELF/NCI analyses suggest that for mechanism (1), where no CO${}_{2}$ is formed, the rate-limiting step corresponds to a late Transition State (TS). On the other hand, for the second mechanism, where concomitant formation of CO${}_{2}$ along the rate-limiting step occurs, an early TS structure is stabilized by the surrounding environment. The inclusion of explicit polarization of the MM environment demonstrated to provide a better representation of the the change density distribution of the QM subsystem, and that it has an important effect on the energetics and profile of the MEP.

## 7. Conclusions

AMOEBA-IL provides accurate results for several ionic liquid systems. Due to the highly charged nature of the ionic liquids, the inclusion of explicit polarization is essential in the description of different properties. Simulations of various IL systems using AMOEBA–IL have resulted in the reproduction of thermodynamic (heat of vaporization, density), transport (diffusion coefficients) and kinetic (water-exchange rates) properties, and prediction of others (phase-changes), that were later corroborated experimentally. However, challenges still remain, for example, in the case of the calculated exchange rate coefficients for the pure water and water/IL systems, the calculated values show deviation with respect to the experimental results, although the trends are well correlated with experiment. AMOEBA-IL’s explicit intra-molecular polarization allowed a better representation of the distribution in charge density due to changes in the configuration of the [SPyr] cation, resulting in a better description of density and diffusion coefficients. Finally, the use of polarization in the MM-environment in QM/MM calculations has proved to be quite important to obtain reliable results in the calculation of energy barriers in systems using IL as solvents. AMOEBA–IL is a multipolar–polarizable potential for ILs that provides accurate description of inter–molecular interactions and bulk properties, and can be used for classical and hybrid QM/MM calculations. Future development of AMOEBA–IL will include expansion of the parameter library and applications to different systems using both classical and QM/MM simulations.

## Figures and Tables

**Table 1:** Calculated ($k_{calc}^{exch}$) and experimental ($k_{exp}^{exch}$) water exchange rates on trivalent lanthanide cations in water, H${}_{2}$O/[EMIm][EtSO${}_{4}$], and H${}_{2}$O/[EMIm][OTf] [103,104].

Solvent	Metal Ion	$k_{calc}^{exch}$, s${}^{-1}$	$k_{exp}^{exch}$, s${}^{-1}$
H${}_{2}$O	Gd${}^{3+}$	1.30 × 10${}^{9}$	8.30 × 10${}^{8}$
H${}_{2}$O	Dy${}^{3+}$	7.72 × 10${}^{8}$	4.34 × 10${}^{8}$
H${}_{2}$O	Ho${}^{3+}$	4.75 × 10${}^{8}$	2.14 × 10${}^{8}$
H${}_{2}$O/[EMIm][EtSO${}_{4}$]	Gd${}^{3+}$	2.96 × 10${}^{7}$	5.08 × 10${}^{6}$
H${}_{2}$O/[EMIm][EtSO${}_{4}$]	Dy${}^{3+}$	4.94 × 10${}^{7}$	3.64 × 10${}^{7}$
H${}_{2}$O/[EMIm][EtSO${}_{4}$]	Ho${}^{3+}$	8.86 × 10${}^{7}$	4.61 × 10${}^{7}$
H${}_{2}$O/[EMIm][OTf]	Gd${}^{3+}$	1.30 × 10${}^{8}$	1.3 × 10${}^{7}$
H${}_{2}$O/[EMIm][OTf]	Dy${}^{3+}$	2.00 × 10${}^{8}$	1.4 × 10${}^{8}$
H${}_{2}$O/[EMIm][OTf]	Ho${}^{3+}$	2.60 × 10${}^{8}$	1.5 × 10${}^{8}$
